# One level up: abnormal proteolytic regulation of IGF activity plays a role in human pathophysiology

**DOI:** 10.15252/emmm.201707950

**Published:** 2017-08-11

**Authors:** Jesús Argente, Julie A Chowen, Luis A Pérez‐Jurado, Jan Frystyk, Claus Oxvig

**Affiliations:** ^1^ Department of Pediatrics & Pediatric Endocrinology Hospital Infantil Universitario Niño Jesús Instituto de Investigación La Princesa Madrid Spain; ^2^ Department of Pediatrics Universidad Autónoma de Madrid Madrid Spain; ^3^ Centro de Investigación Biomédica en Red de Fisiopatología de la Obesidad y Nutrición (CIBEROBN) Instituto de Salud Carlos III Madrid Spain; ^4^ IMDEA Food Institute, CEI UAM + CSIC Madrid Spain; ^5^ Genetics Unit Universitat Pompeu Fabra Barcelona Spain; ^6^ Hospital del Mar Research Institute (IMIM) Barcelona Spain; ^7^ Centro de Investigación Biomédica en Red de Enfermedades Raras (CIBERER) Instituto de Salud Carlos III Barcelona Spain; ^8^ Medical Research Laboratory Department of Clinical Medicine, Health Aarhus University Aarhus C Denmark; ^9^ Department of Molecular Biology & Genetics Aarhus University Aarhus C Denmark

**Keywords:** IGF‐1, IGFBPs, PAPP‐A, PAPP‐A2, stanniocalcins (STC1, STC2), Genetics, Gene Therapy & Genetic Disease

## Abstract

The discovery of a mutation in a specific gene can be very important for determining the pathophysiology underlying the disease of a patient and may also help to decide the best treatment protocol on an individual basis. However, sometimes the discovery of mutations in new proteins advances our comprehension in a more widespread manner. The growth hormone (GH)/insulin‐like growth factor (IGF)‐1 axis is fundamental for systemic growth, but is also involved in many other important processes. Our understanding of this system in physiology and pathophysiology has advanced throughout the years with each discovery of mutations in members of this axis. This review focuses on the most recent discovery: mutations in the metalloproteinase pregnancy‐associated plasma protein‐A2 (PAPP‐A2), one of the proteases involved in liberating IGF‐1 from the complexes in which it circulates, in patients with delayed growth failure. We also discuss the advances in the stanniocalcins (STC1 and STC2), proteins that modulate PAPP‐A2, as well as PAPP‐A. These new advances not only bring us one step closer to understanding the strict spatial and temporal control of this axis in systemic growth and maturation, but also highlight possible therapeutic targets when this system goes awry.

**Glossary**
Acromegaly: Disorder due to an excessive production of growth hormone from an anterior pituitary gland and adenoma leading to overproduction of IGF‐1 resulting in excessive growth of body tissues and other metabolic dysfunctions in adults.
Chondrogenesis: Process of cartilage formation. It is a highly organized multistep process that involves differentiation into chondroblasts and chondrocytes, and the transformation of chondrogenic tissues into skeletal structures.
DXA: “Dual‐energy X‐ray absorptiometry”. Image technology currently employed to measure bone mineral density. The DXA scan is usually employed to diagnose and follow patients with osteoporosis.
Gigantism: A rare disorder caused by excessive GH secretion and high levels of IGF‐1. Gigantism occurs when excess of GH or IGF‐1 leads to increased linear growth, before the end of puberty and epiphyseal closure.
Growth plate: Anatomical structure where the longitudinal growth of long bones and vertebrae occurs.
Laron syndrome: Is an autosomal recessive disorder (MIM # 262500) characterized by severe short stature due to the lack of IGF‐1 in response to GH. This syndrome is caused by mutations in the GH receptor.
Metalloproteinases: A superfamily of metalloproteinases or zinc‐peptidases that share catalytic domain architecture. A class of proteases that requires a metal, such as zinc, for their catalytic action.
Metzincin: Defines a superfamily of zinc‐peptidases.
Skeletal dysplasias: Generic term for all genetic disorders affecting the skeletal growth.

## Introduction

**Figure 1:**
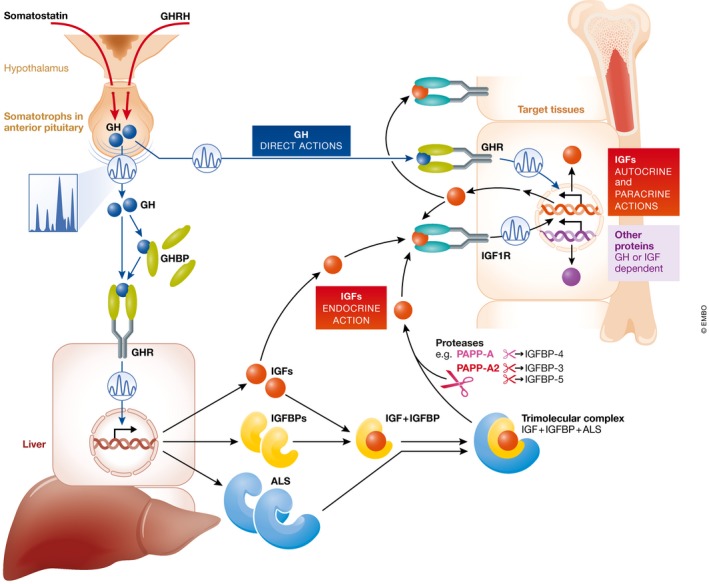
The GH‐IGF axis and its autocrine, paracrine, and endocrine actions GH is secreted in a pulsatile manner from the somatotrophs of the anterior pituitary. In the circulation, GH can be found free or bound to GH binding proteins (GHBP). GH can activate the GH receptor (GHR) directly on target issues (“direct actions of GH”) or stimulate the production of ALS, IGFBPs, and IGFs, which also participate in the promotion of growth. In order to avoid hypoglycemia, IGFs circulate primarily bound to ALS and either IGFBP‐3 or IGFBP‐5 (ternary complex of 150 kDa). Specific proteases, such as PAPP‐A and PAPP‐A2, selectively exert proteolytic activity on IGFBP‐4 or IGFBP‐3 and IGFBP‐5, respectively. Subsequently, free IGF‐1 can activate its receptor on target tissues (“endocrine, autocrine, or paracrine actions” of IGF‐1).

Almost 60 years have passed since Daughaday *et al* (1959) reported the discovery of a *sulfation factor* in patients with pituitary disorders. This factor, first designated as somatomedin C and later as insulin‐like growth factor (IGF)‐1, gained widespread interest due its involvement in promotion of longitudinal growth. However, IGF‐1 is now known to be implicated in numerous processes and our knowledge of the proteins involved in regulating the actions of this growth factor has continued to grow. The majority of circulating IGF‐1 is produced by the liver under the control of growth hormone (GH) released from the anterior pituitary, thus the designation as the GH‐IGF axis (Fig 1). In addition to GH and IGF‐1, the classical members of this axis include IGF‐2, the IGF‐1 receptor (IGF1R), the IGF2 receptor (IGF2‐R), the GH receptor (GHR), the high‐affinity IGF‐binding proteins (IGFBPs) 1–6, and the acid labile subunit (ALS). A large percentage of IGF‐1 circulates bound to one of the six different IGFBPs, principally IGFBP‐3 and IGFBP‐5, fractions of which further form ternary complexes with ALS, resulting in an increase in IGF‐1 half‐life (Baxter, 2000). The IGFBPs circulate in molar excess of IGF‐1 and have ligand affinities that exceed that of the IGF receptor (IGF1R), thus antagonizing the biological activities of the IGFs (Yakar *et al*, 2002).

Although IGF‐1 was first discovered due to the observation that it was able to stimulate longitudinal growth, the list of physiological and pathophysiological processes involving this growth factor is now extensive and includes metabolism, development of specific neurosensory systems, neuroprotection, longevity, cancer, obesity, eating disorders, and neurodegenerative diseases (Argente *et al*, 1997a,b; Katic & Kahn, 2005; Yakar *et al*, 2005; Torres‐Aleman, 2012). Almost all tissues throughout the body not only express receptors for the IGFs, but also produce IGF‐1 (LeRoith & Yakar, 2007), with the correct development and functioning of these tissues depending on the reception of appropriate IGF‐1 signals. Indeed, IGF‐1 is necessary for tissue development and maintenance, but excess activation of the IGF‐1 signaling pathways promotes pathological development, such as gigantism and acromegaly (Hannah‐Shmouni *et al*, 2016). Thus, the IGF signal that a cell receives must be carefully regulated not only in its magnitude, but also temporally. Expression of the IGF‐1R and the IGFBPs is controlled both temporally and spatially, and this is clearly important for the cell‐ and tissue‐specific effects of IGF‐1. However, our understanding of the mechanisms behind this tightly regulated process of cell‐specific actions remains limited. In addition, the more recent discovery of proteases involved in releasing the IGFs from the ternary complex or from the IGFBPs has added a new level of complexity to this system. Here, we discuss how the discovery of a new, hitherto unknown growth‐retarding mutation in a specific protease of the GH‐IGF system has advanced not only our understanding of the physiological growth process, but also exemplifies the intricate control of this system. Excitingly, this novel information may open new avenues of treatment of other IGF pathologies.

## The IGF system in human growth

The regulation of prenatal and postnatal human growth is complex, and we are still in the process of deciphering the molecular mechanisms involved. Many advances have been made during the last two decades in our understanding of the genetics underlying proportionate short stature and in the biochemical comprehension of the GH‐IGF axis; however, our knowledge has remained incomplete regarding the mechanisms controlling liberation of IGFs from their high‐affinity IGFBPs to promote tissue growth in a coordinated and proportionate fashion. Indeed, until recently we had no insight as to the impact of IGFBP‐cleaving enzymes on human postnatal growth. However, the discovery of the first mutations in the metalloproteinase pregnancy‐associated plasma protein‐A2 (PAPP‐A2 or pappalysin‐2) has not only increased our understanding of human postnatal growth, but also our comprehension of the regulation of the GH‐IGF axis. This axis is essential for normal human development, with GH promoting growth both through stimulating the production of hepatic IGF‐1 and by acting directly on the growth plate. Indeed, genetic defects in the GH‐IGF axis result in diverse syndromes that present with impaired growth, and each discovery has added to our understanding of growth physiology (David *et al*, 2011). For example, Laron syndrome, which courses with extreme growth failure, is now known to be the result of mutations in the GHR gene (Amselem *et al*, 1989). Mutations in the genes encoding signal transducer and activator of transcription (STAT)5B (Kofoed *et al*, 2003), one of the proteins in the signaling cascade of the GHR, IGF‐1 (Woods *et al*, 1996), and IGF‐1R (Abuzzahab *et al*, 2003), were subsequently shown to result in pre‐ and postnatal growth retardation of varying degrees, while mutations in the ALS gene cause short stature that can be classified as mild (Domené *et al*, 2004). Two recent reviews detail the advances in the genetic causes of short stature and their clinical features (Argente, 2016; Wit *et al*, 2016).

To date, there has been no report of mutations in any of the genes encoding the six IGFBPs that result in human pathogenesis. It has been speculated to be due to the existence of redundancy in the functions of these IGFBPs, such that no apparent or only a subtle pathological phenotype would result from the functional loss of only one IGFBP. If this is indeed the case, it emphasizes the importance of these regulatory proteins in human physiology. Until recently, there were no known mutations in genes encoding enzymes that regulate the ability of the IGFBPs to interact with IGF‐1, for example*,* PAPP‐A2. Since its discovery, this metalloproteinase has been hypothesized to increase IGF‐1 bioactivity by its ability to specifically cleave IGFBP‐3 and IGFBP‐5, but its significance in the regulation of human growth was unknown. We recently identified a novel autosomal recessive syndrome consisting of short stature, skeletal abnormalities, and high circulating concentrations of IGF‐1, IGFBP‐3, IGFBP‐5, and ALS. This novel syndrome is caused by loss‐of‐function mutations in the gene encoding PAPP‐A2 (Dauber *et al*, 2016). The reported mutations result in dysfunctional PAPP‐A2 or undetectable levels of the proteinase in the circulation. More recently, we demonstrated the efficacy of recombinant human IGF‐1 in improving growth in these patients that lack PAPP‐A2 activity with no apparent adverse effects following 1 year of therapy (Muñoz‐Calvo *et al*, 2016).

PAPP‐A and PAPP‐A2 comprise the only two known members of the pappalysin family of metalloproteinases (Oxvig, 2015). The proteolytic activity of PAPP‐A (pappalysin‐1) was discovered in 1999 (Lawrence *et al*, 1999), and PAPP‐A was later shown to belong to the metzincin class of metalloproteinases; but this protein was still considered to be distinct from previously recognized families, for example, the matrix metalloproteinases (Boldt *et al*, 2001). PAPP‐A2 was discovered in 2001 (Overgaard *et al*, 2001), defining the pappalysin family (Boldt *et al*, 2001). PAPP‐A specifically cleaves IGFBP‐2, IGFBP‐4, and IGFBP‐5 and is now known to be widely expressed in multiple tissues (Oxvig, 2015). It is important to note that IGFBP‐4 only becomes a PAPP‐A substrate once it is bound to IGF‐1 or IGF‐2 (Laursen *et al*, 2001). The cleavage of PAPP‐A results in two proteolytic fragments that show very low affinity for the IGFs, and this results in the dissociation of the IGFBP‐4/IGF complex (Laursen *et al*, 2007). Unlike PAPP‐A2, PAPP‐A tethers to the surface of cells by binding to surface glycosamino glycans (GAGs) (Laursen *et al*, 2002). It is believed that the main function of PAPP‐A is to increase the local bioavailability of IGF by cleaving the inhibitory IGFBPs, especially IGFBP‐4, but this has yet to be supported by genetic evidence in humans. However, PAPP‐A is critical for normal fetal development in mice (Conover *et al*, 2004).

A reduction in IGF signaling has been suggested to increase longevity, as well as health span (Katic & Kahn, 2005). Hence, reducing the proteolytic activity of PAPP‐A, which would indirectly decrease the availability of bioactive IGF, appears to be a potential therapeutic target for healthy longevity (Conover & Oxvig, 2017).

PAPP‐A2 is expressed abundantly in human placenta, in the non‐pregnant mammary gland, and in other tissues, including the kidney, fetal brain, and pancreas (Overgaard *et al*, 2001; Conover *et al*, 2011). Mature PAPP‐A2, which shares 45% of its amino acid residues with PAPP‐A, specifically targets IGFBP‐5 at one site, between Ser143 and Lys144, and also IGFBP‐3 (Overgaard *et al*, 2001). Unlike PAPP‐A, PAPP‐A2 is not membrane bound and does not require the presence of IGF‐1 for its proteolytic function (Overgaard *et al*, 2001).

Recently, stanniocalcin‐1 (STC1) (Kløverpris *et al*, 2015) and stanniocalcin‐2 (STC2) (Jepsen *et al*, 2015) were discovered as potent proteinase inhibitors of PAPP‐A and PAPP‐A2, and accordingly, our knowledge on their connection with the IGF system is novel. While STC2 binds PAPP‐A and PAPP‐A2 irreversibly by the formation of a covalent bond, STC1 binds non‐covalently but with picomolar (pM) affinity. STC1 is abundantly expressed in organs such as kidney, heart, lung, liver, and ovary (Chang *et al*, 1995; Varghese *et al*, 1998) and is reported to be involved in diverse physiological processes including adipogenesis (Serlachius & Andersson, 2004) and chondrogenesis (Wu *et al*, 2006). In addition to growth, STC2 has been implicated in osteoblast differentiation (Zhou *et al*, 2016) and atherosclerosis (Steffensen *et al*, 2016). STC1 knockout mice do not show any abnormalities in growth (Chang *et al*, 2005), while transgenic mice that over‐express STC1 present a severe reduction in growth (Varghese *et al*, 2002). In contrast, increased growth rate results from the absence of STC2 (Chang *et al*, 2008) and severely reduced growth results from overexpression of STC2 (Gagliardi *et al*, 2005); however, mice expressing a mutated form of STC2 (C120A) that is unable to bind PAPP‐A show normal growth (Jepsen *et al*, 2015), suggesting that PAPP‐A action is regulated by STC2 *in vivo*. In a genomewide study by Lango Allen *et al* (2010), SCT2, PAPP‐A, and PAPP‐A2 were all reported to represent loci associated with height. More recently, Marouli and colleagues indicated that STC2 may serve as a brake on human height, suggesting it as a potential drug target for short stature (Marouli *et al*, 2017).

**Figure 2:**
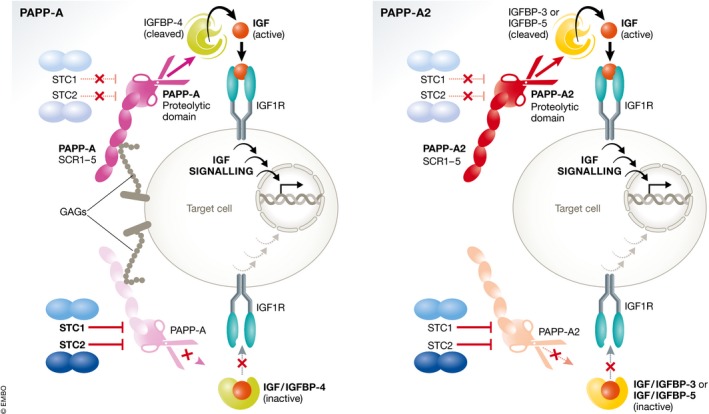
The role of PAPP‐A, PAPP‐A2, STC1 and STC2 in the IGF system The insulin‐like growth factors (IGFs) circulate bound to high‐affinity IGF‐binding proteins (IGFBPs). IGFBP‐4, bound to IGF, can be cleaved by the proteinase pregnancy‐associated plasma protein‐A (PAPP‐A) to liberate IGF, which can then activate its receptor (IGF1R). Stanniocalcin‐2 (STC2) inhibits PAPP‐A proteolytic activity through covalently binding to the proteinase, thus inhibiting the release of IGFs. STC1 is also inhibitory, but binds PAPP‐A reversibly with very high affinity. PAPP‐A2 has proteolytic activity toward IGFBP‐3 and IGFBP‐5. Both STC1 and STC2 can also inhibit the activity of PAPP‐A2, blocking the release of IGFs from IGFBP‐3 and IGFBP‐5. Figure modified from Jepsen *et al* (2015).

A summary of the role of PAPP‐A, PAPP‐A2, STC1, and STC2 can be seen in Fig 2.

## A place for PAPP‐A2 in the clinical setting

The recent discovery and analysis of the first mutations in the human *PAPPA2* gene have increased our understanding of the physiological regulation of the GH‐IGF axis and its activity. The natural history of this discovery started when two children from a non‐consanguineous Spanish family were evaluated at the Department of Pediatric Endocrinology of the University Hospital Niño Jesús in Madrid, Spain, for short stature. The first child, a girl 9 years of age, exhibited a height 1.7 SDS below her mid‐parental height (percentile 50–75); the second child, her younger brother, showed a height 1.3 SDS below his mid‐parental height (percentile 50–75). Interestingly, both children presented very high serum concentrations of IGF‐1, IGF‐2, IGFBP‐3, and ALS (Dauber *et al*, 2016). Spontaneous GH secretion over 8 h was also markedly elevated. Their birth lengths and weights were normal.

Given that the two affected siblings, a female and a male, were from a non‐consanguineous family where the parents were unaffected, we suspected an autosomal recessive inheritance pattern for this disorder. We first excluded mutations in the *IGF1R* gene and then performed whole genome sequencing in the affected female patient. There were five genes that met the established criteria of the presence of a single homozygous or two heterozygous rare (minor allele frequency < 1%) non‐synonymous variants. The novel homozygous frameshift mutation in *PAPPA2* (c.1927_1928insAT, p.D643fs25*) was established as the likely causal variant as the mutation resulted in loss‐of‐function and the encoded protein, PAPP‐A2, had a known role in cleaving IGFBPs and liberating IGF. This variant was confirmed to be homozygous in the affected boy, heterozygous in both parents and in one unaffected sibling, while it was absent in the second unaffected sibling.

Because *Pappa2* gene knockout mice exhibit bone abnormalities (Conover *et al*, 2011), skeletal surveys in the two affected children were performed. They did not show signs of skeletal dysplasia; however, long bones were found to be very thin. In addition, DXA scans identified reduced bone mineral density in the lumbar spine. Later, a second family with similar biochemical and genetic characteristics was identified. In contrast to the Spanish family, this family carried a single‐residue mutation (A1033V) of *PAPPA2* (Dauber *et al*, 2016). This strengthened our demonstration of the new syndrome and confirms, in our view, the intimate involvement of PAPP‐A2 as a regulatory proteinase of the IGF axis and that mutations in the *PAPPA2* gene can result in short stature.

To assess the consequences of the *PAPPA2* mutations at the biochemical level, the variants were expressed recombinantly and compared with wild‐type (WT) PAPP‐A2. When the media from cells that had been transfected with cDNA encoding either of the identified variants was employed, no proteolytic activity toward either IGFBP‐3 or IGFBP‐5 was detected. In contrast, the media from cells that had been transfected with WT *PAPPA2* cDNA were capable of completely cleaving these substrates.

Serum concentrations of bioactive IGF were measured by using the kinase receptor activation (KIRA) assay, which measures the ability of serum to activate the IGF‐1R *in vitro* (Chen *et al*, 2003). As expected, the level of bioactive IGF was very low in both subjects as were free IGF‐1 levels (assessed by immunoassays) and consequently, we hypothesized that low endogenous levels of free/bioactive IGF‐1 were responsible for the poor growth in these children. Most likely, this also explains the reduced ability of circulating IGF‐1 to exert negative feedback inhibition on the pituitary, resulting in increased GH secretion and, in turn, the increased circulating levels of IGF‐1, IGFBP‐3, and ALS. Thus, mutations in *PAPPA2* may not only impact on IGF stimulated growth, but also on the secretion of GH.

## The treatment

In an attempt to improve linear growth in our two Spanish subjects, we administered recombinant human IGF‐1 (rhIGF‐1) to test its effectiveness in promoting at least short‐term growth (Muñoz‐Calvo *et al*, 2016). Initially, 40–80 μg/kg of rhIGF‐1 (Mecasermin, Increlex^®^; Ipsen, Paris, France) was administered subcutaneously twice daily for 6 months. Thereafter, the dose was progressively increased to 120 μg/kg. Of note, the growth response was in the higher range of that observed in patients with GH insensitivity syndrome treated with rhIGF‐1 (Chernausek *et al*, 2007). Treatment of these PAPP‐A2‐deficient patients with rhIGF‐1 also induced modifications in the GH axis that could affect the long‐term growth response. Indeed, the decrease in spontaneous GH secretion resulting from rhIGF‐1 administration could reduce the non‐IGF‐dependent effects of GH on growth. This decline in GH secretion most likely results from an increased negative feedback exerted by the rise in bioactive IGF‐1 after rhIGF‐1 administration (Chen *et al*, 2003).

Treatment with rhIGF‐1 can produce adverse effects, mainly hypoglycemia and disproportionate growth of specific organs (e.g., spleen and kidneys) (Guevara‐Aguirre *et al*, 1995; Chernausek *et al*, 2007), apparently secondary to the marked insulin‐like effect and anabolic effects of high concentrations of IGF‐1 after treatment (Savage, 2013). However, neither of the patients in this study experienced any of these complications. This could be due to the specific abnormalities in their GH‐IGF axis. In fact, both patients have an excess of antagonistic IGFBPs that is not observed in other patients treated with IGF‐1 and we speculate that this may convey protection, at least in part, against the possible adverse rhIGF‐1‐related effects.

The first mutation demonstrated in the *PAPPA2* gene resulted in the description of a new syndrome with: (i) progressive postnatal growth retardation; (ii) markedly elevated circulating concentrations of total IGF‐1, IGF‐2, IGFBP‐3, IGFBP‐5, and ALS, but a decrease in free IGF‐1 levels and IGF bioactivity; (iii) bone abnormalities; and (iv) decreased bone mineral density. Thus, there are new clinical messages derived from what was observed in these patients (Box A).

The long‐term follow‐up of these patients will be of special interest in identifying other possible functions of this specific protease that might be relevant to other actions of IGF‐1 in adults.

**Box A: The clinical take‐home messages.**

- *PAPPA2* gene mutations define a new specific diagnosis in a subset of patients with growth failure that until now were classified as idiopathic.
- In patients with proportionate short stature and elevated IGF‐1 levels, serum PAPP‐A2 levels should be determined.
- In the absence of PAPP‐A2, as well as when subnormal levels are present, molecular studies should be performed.
- The *PAPPA2* gene should be added to the list of genes causing molecular bases of growth failure.
- Prepubertal patients with PAPP‐A2 deficiency can improve their growth by rhIGF‐1 treatment.

## Future directions

Although it has previously been hypothesized that pappalysins were important regulators of IGF activity in humans, the patients lacking PAPP‐A2 have now demonstrated that this is indeed the case. The identification of PAPP‐A mutations, as well as mutations in the STCs, the regulators of these proteinases, would also verify the hypothesis that both pappalysins and their regulators are intimately involved in the regulation of human growth. Indeed, rare variants of STC2, showing slightly compromised ability to inhibit PAPP‐A (Marouli *et al*, 2017) and PAPP‐A2 (unpublished), were recently identified. This finding suggests that in normal physiology, STC2 serves as a *brake* on human height and therefore that STC2 may be a potential drug target to correct growth retardation (Marouli *et al*, 2017); disabling or antagonizing STC2 activity is likely to increase the activity of PAPP‐A and PAPP‐A2 with the effect of causing increased IGF signaling. Although IGF‐1 treatment for patients with low IGF bioactivity is a more direct approach, the advantage of using agents that antagonize the inhibitory activity of STC2 would be a reduction in the frequency of injections, as IGF‐1 treatment generally requires at least two daily injections, and the possible adverse long‐term effects of IGF‐1 treatment could be avoided. Thus, more information is necessary regarding the human physiology of these proteins and their possible use as therapeutic targets.

Little is known regarding how the expression of these regulatory proteins is controlled or whether they are differentially expressed in tissues at different times during development. For example, it remains unknown whether pappalysins and stanniocalcins play a role in the pubertal growth spurt. Are they under nutritional control? How are they affected by stress? Indeed, it is enticing to hypothesize that any factor that affects human growth and development could be mediating some of their effects through the network of these regulatory proteins. For example, a seemingly minor shift in the balance between active and inhibited PAPP‐A/PAPP‐A2 may have a large effect of the generation of bioactive IGF‐1 locally or systemically. Thus, there is an exciting new area of research that could help us to understand, and improve, human growth and development. Of importance, this would also be relevant to multiple other processes of normal and pathological human physiology, for example, neurodegenerative diseases, aging, and cancer, where aberrant IGF signaling has been implicated but remains only partially understood.

As the IGF system is involved in many processes throughout life, understanding its cell‐/tissue‐specific regulation is fundamental in order to target this growth factor in treatment of specific diseases. For example, IGF‐I plays a protective role against neurodegenerative diseases (Torres‐Aleman, 2012). One might envision the possibility of increasing the central actions of IGF‐I through activation of specific proteases involved in the liberation of IGFs to specific neuronal populations. However, much is yet to be learned regarding the cell specificity of this system, but with the identification of these new members of this axis, we have hopefully come at least one step closer to understanding the physiology, and hence pathophysiology, of this system.

To arrive to another level in our understanding of this system, clinical and basic researchers must continue working closely together to achieve advances that will result in new diagnoses and potentially new treatments for our patients.

**Pending issues**

- Demonstration that mutations in the *PAPPA* gene affect human growth.
- Demonstration that mutations in the stanniocalcins affect human physiology.
- Treatment of patients with *PAPPA2* mutations with recombinant PAPP‐A2.
- Determination of the physiological response to inhibitors of stanniocalcin 2.
- Analysis of changes in pappalysins and stanniocalcins in puberty.
- Study of the physiological control of expression of pappalysins and stanniocalsins.
- Determine the possible role of pappalysins and stanniocalsins in pathology, including cancer and neurodegenerative diseases.

## Conflict of interest

The authors declare that they have no conflict of interest.
